# Oral Vaccination with Heat Inactivated *Mycobacterium bovis* Activates the Complement System to Protect against Tuberculosis

**DOI:** 10.1371/journal.pone.0098048

**Published:** 2014-05-19

**Authors:** Beatriz Beltrán-Beck, José de la Fuente, Joseba M. Garrido, Alicia Aranaz, Iker Sevilla, Margarita Villar, Mariana Boadella, Ruth C. Galindo, José M. Pérez de la Lastra, Juan A. Moreno-Cid, Isabel G. Fernández de Mera, Pilar Alberdi, Gracia Santos, Cristina Ballesteros, Konstantin P. Lyashchenko, Esmeralda Minguijón, Beatriz Romero, Lucía de Juan, Lucas Domínguez, Ramón Juste, Christian Gortazar

**Affiliations:** 1 SaBio IREC (CSIC-UCLM-JCCM), Ciudad Real, Spain; 2 Department of Veterinary Pathobiology, Center for Veterinary Health Sciences, Oklahoma State University, Stillwater, Oklahoma, United States of America; 3 NEIKER-Tecnalia, Animal Health Department, C/Berreaga 1, Derio, Bizkaia, Spain; 4 Dept. Sanidad Animal, Facultad de Veterinaria, Universidad Complutense de Madrid, Madrid, Spain; 5 Chembio Diagnostic Systems Inc., Medford, New York, United States of America; 6 Centro de Vigilancia Sanitaria Veterinaria (VISAVET), Facultad de Veterinaria, Universidad Complutense de Madrid, Madrid, Spain; University of Padova, Medical School, Italy

## Abstract

Tuberculosis (TB) remains a pandemic affecting billions of people worldwide, thus stressing the need for new vaccines. Defining the correlates of vaccine protection is essential to achieve this goal. In this study, we used the wild boar model for mycobacterial infection and TB to characterize the protective mechanisms elicited by a new heat inactivated *Mycobacterium bovis* vaccine (IV). Oral vaccination with the IV resulted in significantly lower culture and lesion scores, particularly in the thorax, suggesting that the IV might provide a novel vaccine for TB control with special impact on the prevention of pulmonary disease, which is one of the limitations of current vaccines. Oral vaccination with the IV induced an adaptive antibody response and activation of the innate immune response including the complement component C3 and inflammasome. Mycobacterial DNA/RNA was not involved in inflammasome activation but increased C3 production by a still unknown mechanism. The results also suggested a protective mechanism mediated by the activation of IFN-γ producing CD8+ T cells by MHC I antigen presenting dendritic cells (DCs) in response to vaccination with the IV, without a clear role for Th1 CD4+ T cells. These results support a role for DCs in triggering the immune response to the IV through a mechanism similar to the phagocyte response to PAMPs with a central role for C3 in protection against mycobacterial infection. Higher C3 levels may allow increased opsonophagocytosis and effective bacterial clearance, while interfering with CR3-mediated opsonic and nonopsonic phagocytosis of mycobacteria, a process that could be enhanced by specific antibodies against mycobacterial proteins induced by vaccination with the IV. These results suggest that the IV acts through novel mechanisms to protect against TB in wild boar.

## Introduction

Tuberculosis (TB) caused by members of the *Mycobacterium tuberculosis* complex affects more than 2.5 billion people worldwide with approximately 9 million new cases reported every year [1]. The Bacillus Calmette-Guérin (BCG) vaccine has been widely used for TB control [2]. However, BCG and other first-generation vaccines do not prevent infection nor achieve sterile eradication, but rather prime and/or boost infection control. Therefore, the development of new safe vaccines is required to achieve full protection in some areas and age groups, and particularly to protect against pulmonary disease and infection rather than from active TB [2], [3]. Additionally, the correlates of protection for TB and BCG vaccines are poorly defined and constitute essential information for the development of improved vaccines [2], [4]–[6].

Recently, we developed a model for mycobacterial infection and TB using Eurasian wild boar (*Sus scrofa*) [7], [8]. Wild boar are susceptible to mycobacterial infection and reproduce some of the clinical symptoms observed in human TB cases such as lung pathology and latent infection [9], [10]. Molecular characterization of host-pathogen interactions identified *S. scrofa* genes such as methylmalonyl CoA mutase (*MUT*), complement component 3 (*C3*) and other innate and adaptive immune response genes involved in resistance to mycobacterial infection [11]–[16].

Wild boar are natural reservoir hosts for *Mycobacterium bovis* in some regions and thus vaccination strategies are being developed for TB control in this species [7], [8], [10]. Recently, parenteral and oral vaccination with a heat-inactivated *M. bovis* vaccine (IV) protected wild boar against TB with special reduction in thorax tuberculous lesions [8]. These results suggested that oral vaccination with the IV might constitute a novel approach for TB control with the aim of preventing or drastically reducing acquisition and establishment of infection. However, as for other vaccines for TB control, the protection mechanisms elicited by the IV remain unclear and are the focus of this study. In these experiments, we did not focus on a preconceived mechanism, but rather explored the hypothesis that different mechanisms including adaptive and innate immune responses may constitute possible correlates of protection for the IV.

## Materials and Methods

### Ethics Statement

Animals were monitored daily by the veterinarians. Handling procedures and sampling frequency were designed to reduce stress and health risks for subjects, according to European (86/609) and Spanish legislation (R.D. 223/1988, R.D. 1021/2005). For oral vaccination, challenge and bleeding, restraint was not longer than 10 minutes/animal. When required in nervous or stressed animals, wild boar and pigs were anesthetized prior to bleeding with tiletamine-zolazepam (TZ) (3 mg/kg) and medetomidine (M) (0.05 mg/kg). At the end of the experiment, animals were anesthetized with the protocol described above followed by the use of the captive bolt method.

During the experiment mini pigs were group housed in the Biosafety Level 3 containment of the Animal Health Surveillance Centre (VISAVET, Complutense University of Madrid, Spain). The protocol was approved by the “Comunidad de Madrid” IACUC (Regional agriculture authority; permit number: CM180112-01 (18/01/2012)). Wild boar were located in one group in the Biosafety Level 3 containment of the Basque Institute for Agricultural Research and Development (NEIKER-Tecnalia) and the protocol was approved by the Committee on the Ethics of Animal Experiments of the Regional Agriculture Authority (Diputación Foral de Vizcaya, Permit Number: BFA10.373 (27/19/2010)).

### Preparation of the IV

The *M. bovis* field isolate Neiker 1403 (spoligotype SB0339) originally obtained from a naturally infected wild boar was used for IV preparation. The isolate was cultivated for 2–3 weeks in Middlebrook 7H9 medium enriched with OADC. Cells were obtained after centrifugation at 2,500×g for 20 minutes at room temperature (RT) and after two washes in PBS, the pellet was resuspended in PBS and passed through an insulin syringe for declumping. The optical density of the suspension was adjusted with PBS to 5 McFarland units. Prior to inactivation, serial dilutions (x10) were seeded in quadruplicate on 7H9 plates with OADC solidified with agar in order to quantify the number of colony forming units (cfu) in the inoculum. The inoculum was inactivated in a shaking water bath at 81–83°C for 40 minutes. Inactivated inoculum was cultured in BACTEC Mycobacterial Growth Indicator Tubes supplemented as indicated by the manufacturer (Becton Dickinson) and onto OADC agar solidified 7H9 plates in triplicate (100 µl each) to confirm the absence of viable mycobacteria. The final IV preparation contained approximately the equivalent of 10^7^ cfu in 0.2 ml of PBS.

### Purification and Characterization of Nucleic acids from the IV

One ml of IV was pelleted and resuspended in 500 µl of Tri Reagent (Sigma-Aldrich St. Louis MO, USA). The solution was sonicated during 1 hr in 6 cycles of 10 minutes each and then immediately subjected to continuous vortex mixing for 30 min [17]. The lysate was left at room temperature for 4 hrs with intermittent vortex mixing every 15 min. Total RNA was extracted following manufacturer instructions. Two ml of IV were treated with 40µl (80 units) of DNaseI in 0.2 ml of 10X DNase I Buffer (Ambion Life Technologies, Grand Island, NY, USA) and incubated at 37°C for 1 hr. Six ml of water and 0.8 ml of 10x RNAse ONE buffer were then added in order to provide suitable conditions for the RNAse ONE Ribonuclease enzyme (Promega, Madison WI, USA). The reaction was incubated at 75°C for 10 min. Subsequently, 95 µl of RNAse ONE (10 units/µl) were added to the reaction and incubated at 37°C for 3 hrs. Total RNA was used to synthesize cDNA using random primers and the Access RT-PCR system (Promega, Madison, WI, USA). Total RNA and IV samples were characterized by RT-PCR using oligonucleotide primers Mycgen-F: 5′-AGAGTTTGATCCTGGCTCAG-3′ and Mycgen-R: 5′-TGCACACAGGCCACAAGGGA-3′ that amplify 16S rRNA from *Mycobacterium tuberculosis* complex [18] and the Access RT-PCR system (Promega, Madison, WI, USA). Amplicons were cloned and six independent clones were sequenced for analysis. The evolutionary history was inferred using the Neighbor Joining method [19] in MEGA5 (http://www.megasoftware.net). Blasting against nonredundant sequence database (nr) was done using blastn (http://blast.ncbi.nlm.nih.gov/Blast.cgi). Nucleotide sequences were aligned using the program AlignX (Vector NTI Suite V 5.5, InforMax, North Bethesda, MD, USA).

To characterize the effect of mycobacterial DNA/RNA in the IV, four mini pigs were randomly allocated to two groups of two animals each and orally vaccinated at days 1 (T0) and 28 with 2 ml of the IV depleted of mycobacterial DNA and RNA (IV-DNA/RNA) and IV. One month after last vaccination (T1), animals were challenged with 5 ml of a suspension containing 10^5 ^cfu of an *M. bovis* field strain and then euthanized four months after challenge (T2) [7], [8]. Oral mucosa and blood samples were collected to compare oral mucosa and peripheral blood mononuclear cells (PBMC; Buffy coat) *C3* mRNA levels and serum C3 protein and cytokine levels between animals vaccinated with IV and IV-DNA/RNA.

### Wild Boar Vaccination and Challenge

Fifteen 3–4-month-old wild boar piglets were bought in a commercial farm known to be free of mycobacterial lesions at slaughter and with a fully negative ELISA test [20]. The animals were housed in class III bio-containment facilities where they had ad libitum food and water. Wild boar piglets were randomly assigned to two treatment groups, vaccinated (N = 7) and control (N = 8). The IV (10^7^ cfu) was delivered in baits designed for wild boar piglets [21]. One bait per individual was introduced by hand into the oral cavity, making sure the animal chewed and opened the vaccine-containing vial. For challenge, 5 ml of a suspension containing 10^5 ^cfu of an *M. bovis* field strain (spoligotype SB0339) were administered by the oropharyngeal route as described in previous experiments [7], [8]. The animals were vaccinated at T0, revaccinated at day 52, challenged at day 126 (T1) and necropsied at day 255 (T2).

### Sample Collection, Necropsy and Histopathology

Blood samples were collected at T0, T1 and T2 time points for RNA extraction from PBMC and serum preparation. Animals were anesthetized by intramuscular injection of zoletil, and euthanized by captive bolt. A thorough postmortem examination was done to detect the presence of macroscopic lesions. Samples for culture were immediately processed and copies frozen at −80°C for RNA isolation. Tonsils, lymph nodes, lung (each lobe considered separately), spleen, and ileocecal valve were carefully inspected for TB-compatible lesions and cultured. When suspicious lesions were observed in liver, kidney and lymph nodes from other locations, samples from these tissues were also cultured. TB-compatible lesions were classified based on lesion distribution and lesion intensity, and scored as previously described [7]. Samples of individual tissues were fixed in 10% buffered formalin, embedded in paraffin, sectioned at 4 µm, and stained with hematoxylin–eosin by use of standard procedures. Additional sections of tissues with lesions indicative of TB were stained by Ziehl–Neelsen procedure to detect the presence of acid-fast organisms.

### Microbiology

All samples were cultured and spoligotyped in order to confirm the strain as previously described [8]. We defined a culture score for *M. bovis* infection as the number of lymph nodes or organ samples yielding a *M. bovis* isolate, of the total number of culture attempts (N≥17 samples cultured per animal; score range 0–17). Infection levels in different samples were categorized according to the number of colonies per tube as follows: less than 10 colonies, between 10 and 50 colonies and more than 50 colonies [8].

### Serological Analyses

#### Antibody response

Serum samples were tested for antibodies against purified bovine tuberculin protein derivative (bovine PPD; CZ Veterinaria, Porriño, Spain) using an in-house ELISA with Protein G (Calbiochem, Merck KGaA, Darmstadt, Germany) conjugate as described by Boadella et al. [20]. Samples with E% (E% = (sample O.D._450 nm_/2×mean negative control O.D._450 nm_)x100) >100 were considered positive. Antibodies against mycobacterial GAPDH, NADPAD, MPB83, Rv2623 recombinant proteins were tested with the same ELISA described before but coating the plates with 1 µg/well. DPPBovidTB and DPPVetTB were used to test serum samples against DPP as previously described [22]. The presence and intensity of either the sole T band (a mixture of MPB70 and MPB83 proteins) of the DPPBovidTB or the 2 separate test bands (T1, MPB83 antigen; T2, CFP10/ESAT-6 fusion protein) of the DPPVetTB, were evaluated by a DPP optical reader (in relative light units, RLU). Reactivity above the cut-off value of 5.0 RLU in any of the test bands was considered a positive result for the presence of antibody [23].

#### Complement component 3

For the quantitative determination of pig C3 protein concentration in serum samples, a commercially available sandwich ELISA was used (Pig Complement C3 ELISA kit, CUSABIO, Wuhan, China). Serum samples and standards were analyzed following the manufacturer’s instructions. Data was linearized by a standard curve and regression analysis was used to determine sample C3 concentrations in µg/ml.

#### Cytokines

The cytokine concentration in pooled sera was determined at T0, T1 and T2 using the Quantibody porcine cytokine array (RayBiotech Inc, Norcross, GA, USA), an array-based multiplex ELISA system for the simultaneous quantitative measurement of multiple cytokines. Using this system, standard cytokines and samples were assayed in each array simultaneously through a sandwich ELISA procedure, following the recommendations of the manufacturer. The signals were visualized using a Gene Pix 4100A laser scanner (Molecular Devices, Sunnyvale, CA, USA) and data were extracted by GenePix Pro 6 software (Molecular Devices). Finally, the quantitative data analysis was performed using the Quantibody Q-Analyzer software (RayBiotech Inc). Cytokine concentration was expressed in pg/ml and two replicates were performed for each sample.

### Identification of Mycobacterial Proteins Recognized by Vaccinated Animals

The IV proteins were analyzed using denaturing SDS-PAGE with a 12% PAGEgelTM SDS cassette gel (PAGE-gel Inc., San Diego, CA, USA) under reducing conditions. The bands were visualized by Expedeon’s InstantBlue staining. Electrophoretic transfer of proteins from gels to nitrocellulose membranes (Schleicher & Schuell, PROTRAN BA85, Dassel, Germany) for Western blot analysis was carried out in a Minie-Genie Electroblotter semi-dry transfer unit (Idea Scientific, Corvallis, OR, USA) according to the manufacture’s protocol. Membranes were blocked with 5% (w/v) skim milk overnight at 4°C, and then washed three times in TBS (100 mM Tris-base, 150 mM NaCl, pH 7.5) and incubated for 1 hr with boar sera (1∶200 dilution in TBS). Membranes were then washed three times with TBS and incubated with anti-pig IgG peroxidase conjugate (Sigma-Aldrich, St. Louis, MO, USA) diluted 1∶1000 for 1.25 hr at room temperature. After washing the membranes with TBS, color was developed using tetramethylbenzidine (TMB)-stabilized substrate for horseradish peroxidase (Promega, Madison, WI, USA).

Proteins recognized by sera from animals vaccinated with the IV were visualized by Coomassie Brilliant Blue R-250 staining, excised, cut into cubes (2×2 mm) and digested overnight at 37°C with 60 ng/µl trypsin (Promega, Madison, WI, USA) at 5∶1 protein:trypsin (w/w) ratio in 50 mM ammonium bicarbonate, pH 8.8 containing 10% (v/v) acetonitrile [24]. The resulting tryptic peptides from each band were extracted by 30 min incubation in 12 mM ammonium bicarbonate, pH 8.8. Trifluoroacetic acid was added to a final concentration of 1% and the peptides were finally desalted onto C_18_ ProteaTips (Protea Biosciences, Inc., Morgantown, WV, USA), dried-down and stored at 20°C until mass spectrometry analysis. The protein digest was resuspended in 0.1% formic acid and analyzed by RP-LC-MS/MS using a Surveyor LC system coupled to an ion trap LCQ Fleet mass spectrometer (Thermo Scientific, San Jose, CA, USA). The LCQ was programmed to perform a data-dependent MS/MS scan on the 3 most intense precursors detected in a full scan from 400 to 1600 amu, using an isolation width of 3 amu, normalized collision energy of 30%, and dynamic exclusion applied during 3 min periods. Protein identification was carried out using the SEQUEST algorithm (Proteome Discoverer 1.1, Thermo Scientific). Database search was performed against the Actinobacteria database downloaded from the Protein Knowledgebase (UniProtKB; http://www.uniprot.org). The following constraints were used for the searches: tryptic cleavage after Arg and Lys, up to two missed cleavage sites, and tolerances of 1 Da for precursor ions and 0.8 Da for MS/MS fragment ions and the searches were performed allowing optional Met oxidation and Cys carbamidomethylation. A 1% false discovery rate (FDR) was the criteria used for acceptance of peptides assignments and subsequent protein identification.

### Cloning, Expression and Characterization of *M. bovis* Recombinant Proteins

The genes encoding for proteins identified by Western blot and proteomics analysis were amplified by PCR (Table 1), sequenced, cloned into the pTXB1 expression vector (New England Biolabs, Hitchin, Hertfordshire, UK) for expression in *Escherichia coli*. Three independent clones were sequenced for analysis blasting against nonredundant sequence database (nr) using blastn (http://blast.ncbi.nlm.nih.gov/Blast.cgi). For production of recombinant proteins, each strain was propagated overnight in 10 ml cultures in 50-ml shaker flasks at 37°C. The cells were harvested by centrifugation at 3900 × *g* for 15 min at 4°C and cell samples of 1 g wet weight were resuspended in 5 ml of lysis buffer (100 mM NaH_2_PO_4_, 10 mM Tris–HCl, 8 M Urea, pH 7.0) and disrupted by sonication (Bandelin Sonoplus, Berlin, Germany). The cell lysate was dialyzed against column buffer (100 mM NaH_2_PO_4_, 10 mM Tris–HCl, 4 M Urea, pH 7.0) and centrifuged at 10,000×g for 15 min at 4°C. The cell debris was discarded and the clarified cell extract was loaded into chitin-affinity purification columns (New England Biolabs). The elution was carried out with 3 column volumes of cleavage buffer (column buffer containing 50 mM DDT) for 16 h at 4°C. The eluted fractions were dialyzed in PBS, pH 8.0 to remove the excess of thiol reagent. Recombinant proteins were analyzed by SDS-PAGE and Western blot as previously described.

**Table 1:** Oligonucleotide primers and PCR conditions for *M. bovis* (IV isolate) genes.

Gene (Genbankaccession number)	Upstream/downsteam primer sequences (5′-3′)	PCR annealing temperature	PCR product size (bp)
NADP-dependent alcoholdehydrogenase C(NADPAD) (P0A4X1)	GGTGGTCATATGAGCACTGTTGCCGCCTAC/GGTGGTTGCTCTTCCGCACAGGGCTGAGATGTCGATGAC	56°C	1,041
glyceraldehyde-3-phosphate dehydrogenase(GAPDH) (P64179)	GGTGGTCATATGGTGACGGTCCGAGTAGGCATC/GTGGTTGCTCTTCCGCAGAGCGACTTGCCGACCAGCGT	66°C	1,023
MPB83 (ACD61707)	GGTGGTCATATGATCAACGTTCAGGCCAAA/GGTGGTTGCTCTTCCGCACTGTGCCGGGGGCATCAGCAC	42°C	663
Universal stress protein RV2623/MT2698 (NP_337200)	GGTGGTCATATGTCATCGGGCAATTCATCT/GGTGGTTGCTCTTCCGCAAGTCAGCGACTCGCGTGCCAC	42°C	894

### *In vitro* Experiments

#### *In vitro* infection and analysis of M. bovis

The pig lung alveolar macrophage cell line 3D4/31 [25] was kindly donated by Juan José Garrido Pavón (University of Córdoba, Spain) and maintained at 37°C in a 5% CO_2_ atmosphere in RPMI 1640 supplemented with 10% FCS and 0.1 mM Non-essential amino acids (Life Technologies, Paisley, UK). Approximately 2×10^6^ cells were experimentally infected with *M. bovis* (SB0339 strain) at 5 multiplicities of infection (5 mycobacteria per cell). After a 3 h incubation, free bacteria were removed by washing with PBS, then cells were incubated for 48 h with different concentrations of C3 (without C3, 0 µg/ml; low C3 concentration, 26 µg/ml; and high C3 concentration, 105 µg/ml) in the presence of 10% sera from control wild boar, IV-vaccinated wild boar and rabbit IgGs raised against recombinant mycobacterial proteins MPB83 and NADPAD. All treatments were tested in duplicate. Nucleic acids from cell cultures at zero and 48 h post infection were extracted with Tri Reagent (Sigma Aldrich, St. Louis, MO) following manufacturer’s recommendations. DNA was used to determine mycobacterial infection levels by real-time PCR using two different targets. The major immunogenic protein *MPB70*, a highly conserved gene within the *M. tuberculosis* complex, was amplified using primers mpb70-F (5′-CTCAATCCGCAAGTAAACC-3′) and mpb70-R (5′-TCAGCAGTGACGAATTGG-3′). The insertion sequence *IS6110* was amplified using the commercial TaqVet *M. tuberculosis* Complex Kit (LSI, Lissieu, France). The DNA values were normalized against *S. scrofa* cyclophilin using the genNorm ddCT method [26].

#### Characterization of gene expression in pig DCs

Blood samples were collected from the retro-orbital venous sinus of healthy pigs in tubes with heparin. PBMC were purified by Lymphoprep (Axis-shield, Oslo, Norway) density gradient centrifugation as previously described [27]. Briefly, blood was diluted 1∶2 in PBS (Life Technologies, Paisley, UK) and after centrifugation at 400×g for 40 min, the mononuclear cell fraction was washed three times with PBS and then resuspended in RPMI 1640 medium (Life Technologies) supplemented with 20 mM HEPES buffer, l-glutamine (2 mM), penicillin (200 IU/ml), streptomycin (100 µg/ml), 2-mercaptoethanol (5×10^−5^ M) and 5% foetal calf serum (Life Technologies). 5×10^6^ cells/ml were seeded into 25-cm^3^ flasks (Thermo Scientific, Rockford, IL, USA) at 7 ml/flask. The flasks were incubated at 37°C for 3 h and nonadherent cells were removed. The adherent cell population was rinsed gently with medium before fresh medium with cytokines was added. The cytokines used were recombinant swine proteins GM-CSF at 50 ng/ml, IL-4 at 50 ng/ml and IFN-α at 0.8 U/ml (Invitrogen, Carlsbad, CA, USA). Cytokines were added on the first day of culture and one third of the culture volumes were replaced every second day. After 5 days of culture the majority of the cells had obtained a dendritic morphology. The cells were then removed from the flasks by 5–15 min incubation on ice, washed 1–2 times with culture medium and aliquoted in 24 well plates (7×10^5^ cells/well). DCs were incubated for 48 h with different amounts of the IV (0, 10^6^, 5×10^6^, 10^7^, 2×10^7^, and 4×10^7^ cfu/well; N = 6 per treatment). After incubation, cells were collected, washed with PBS and total RNA extracted for gene expression analysis by real-time RT-PCR.

### RNA Isolation and Real Time RT-PCR

Total RNA was extracted from wild boar and pig PBMC, pig oral mucosa and DCs using TRI reagent (Sigma, Madrid, Spain) and the RNeasy kit (Qiagen, Izasa, Madrid, Spain), respectively following manufacturer’s recommendations. RNA was used for real-time RT-PCR analysis of mRNA levels of selected genes in individual samples. Selected genes are involved in innate immunity (complement component 3, *C3*; NLR family, pyrin domain containing 3, *NLRP3*; Toll-like receptor adaptor molecule 1, *TRIF*; myeloid differentiation primary-response protein 88, *MYD88*; interleukin 1-beta, *IL-1b*; interferon gamma, *IFN-γ*; and interferon beta, *IFN-b*), mucosal immunity (Perforin, *PERF* and chemokine (C-C motif) receptor 7, *CCR7*), and methylmalonyl CoA mutase, *MUT* (Table 1). Real-time RT-PCR was performed with gene-specific primers (Table 2) using the One-Step RT-PCR Kit with SYBR Green and the CFX thermal cycler (Bio-Rad, Hercules, CA, USA) following manufacturer’s recommendations. Control reactions were performed using the same procedures, but without RT to monitor DNA contamination in the RNA preparations and without RNA added to monitor contamination of the PCR reaction. A dissociation curve was run at the end of RT-PCR reaction to ensure that only one amplicon was formed and that the amplicon denatured consistently at the same temperature range for every sample [28]. The mRNA values were normalized against *S. scrofa* cyclophilin, β-actin and GAPHD using the genNorm ddCT method [26].

**Table 2:** Oligonucleotide primers and RT-PCR conditions for the analysis of mRNA levels.

GenBank accesion number	Gene	Primer sequences (5′- 3′)	PCR annealing conditions
NM_214009	Complement component 3,	SsC3-L: acaaattgacccagcgtagg	55°C, 30s
	C3	SsC3-R: gcacgtccttgctgtactga	
DQ913893	Interferon gamma, IFN-γ	SsIFNg-L: ttcagctttgcgtgactttg	55°C, 30s
		SsIFNg-R: tcctttgaatggcctggtta	
NM_214055	Interleukin 1-beta, IL-1β	SsIL1beta-L: ccaaagagggacatggagaa	55°C, 30s
		SsIL1beta-R: ttatatcttggcggcctttg	
NM_214405	Methylmalonyl CoA	SsMUT-L: gtttgccaacggtgaaaagt	55°C, 30s
	mutase, MUT	SsMUT-R: aatgagcttcaaggcagcat	
JN391525	Interferon beta, IFN-β	Ss-IFNBF: tcagaagctcctgggacagt	55°C, 30s
		Ss-IFNBR: atctgcccatcaagttccac	
AY373815	Perforin 1, PRF1	Ss-PerfF: gctccaccctgagttcaaga	57°C, 30s
		Ss-PerfR: agtcctccacctccttggat	
NM_001001532	Chemokine (C-C motif)	Ss-CCR7F: tgtgcttcaagaaggacgtg	57°C, 30s
	receptor 7, CCR7	Ss-CCR7R: aagggtcaggaggaagagga	
KF280280	Toll-like receptor adaptor	Hs-TRIFF: caggagcctgaggagatgag	55°C, 30s
	molecule 1, TRIF	Hs-TRIFR: ctgggtagttggtgctggtt	
KF280281	NLR family, pyrin domain	Hs-NLRP3F: cttctctgatgaggcccaag	55°C, 30s
	containing 3 (NLRP3)	Hs-NLRP3R: gcagcaaactggaaaggaag	
NM_001206359	Glyceraldehyde-3-	Ss-GAPHDF: gtcggttgtggatctgacct	55°C, 30s
	phosphate dehydrog., GAPHD	Ss-GAPHDR: agcttgacgaagtggtcgtt	
EU077229	Myeloid differentiation	Ss-MyD88F: cggaggagatgaacttcgag	56°C, 30s
	Prim.-respon. prot. 88, MYD88	Ss-MyD88R: actttcggcagtcctcttca	
DQ452569	β-actin	Ss-BactinF: ggacctgaccgactacctca	55°C, 30s
		Ss-BactinR: ggcagctcgtagctcttcat	
AY008846	Cyclophilin	SsCyclophilin-L: agcactggggagaaaggatt	55°C, 30s
		SsCyclophilin-R: cttggcagtgcaaatgaaaa	

### Flow Cytometry

Not coagulated wild boar whole blood (100 µl) was stained simultaneously with 1 µg each of FITC-conjugated anti-pig CD4 and PE-conjugated anti-pig CD8 monoclonal antibodies (BD Biosciences, Madrid, Spain) following the manufacturer recommendations. After staining, erythrocytes were lysed using BD FACS Lysing Solution (BD Biosciences) and washed. Flow cytometry was performed on a FACScalibur flow cytometer (BD Biosciences). Peripheral blood leukocytes were identified by their characteristic scatter profile. The CD4+/CD8+ ratio was calculated as the number of CD4+ positive cells versus the number of CD8+ positive cells.

### Statistical Analyses

Lesion scores, culture scores, mRNA levels (normalized Ct values), antibody levels (D.O._450 nm_), C3 protein levels (µg/ml), cytokine levels (pg/ml) and CD4+/CD8+ and CD8+/CD4+ T cell ratios at T1 were compared between groups by Student’s t-test with unequal variance (P = 0.05). *M. bovis* infection levels (normalized DNA Ct values) were compared between 0 and 48 h for each treatment by χ2-test (P = 0.05). Regression was used to analyze the relation between lesion and culture scores and serum antibody levels or *C3* mRNA and serum protein levels. The significance of the correlation (95% C.I.) was calculated using Simple Interactive Statistical Analysis (SISA; http://www.quantitativeskills.com/sisa/statistics/corrhlp.htm).

## Results and Discussion

### Oral Vaccination with the IV Protects Boar against Mycobacterial Infection

**Figure 1:**
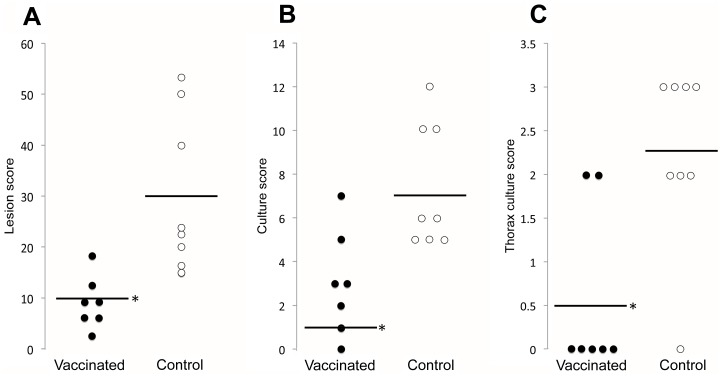
Oral vaccination with IV protects wild boar against mycobacterial infection. (A) Lesion score, (B) culture score and (C) thorax culture score in vaccinated (N = 7) and control (N = 8) wild boar. Solid lines show the median values. Values were compared between groups by Student’s t-test with unequal variance (*P≤0.005).

Oral vaccination with the IV resulted in significantly (P<0.005) lower lesion and culture scores in vaccinated wild boar (Figs. 1A and 1B). The most dangerous and contagious mycobacterial infection is located in the thorax and associated with pulmonary disease. Thorax culture score was significantly lower (P<0.005) in vaccinated than in control animals with high proportion of vaccinated animals with zero culture score (71% versus 13% in control animals) (Fig. 1C). These results suggested that the IV might provide a novel vaccine for TB control with special impact on the prevention of pulmonary disease, which is one of the limitations of current vaccines [3].

### Oral Vaccination with the IV Induces Antibody Response

**Figure 2:**
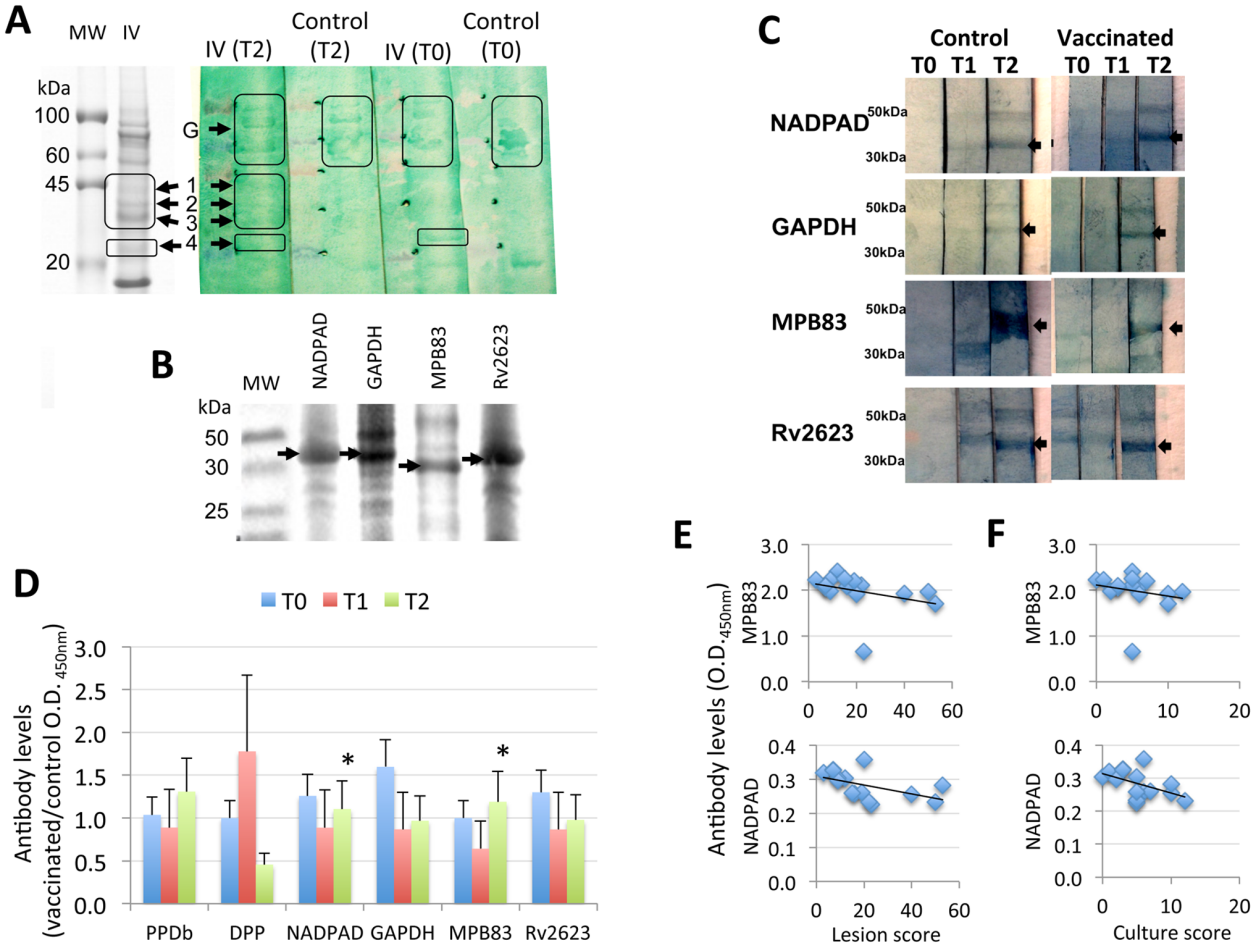
Oral vaccination with IV induces an antibody response in wild boar. (A) Identification by Western blot and mass spectrometry of mycobacterial proteins recognized by antibodies in IV vaccinated wild boar. Identified proteins are indicated with arrows in Coomassie-blue stained 12% SDS-polyacrylamide gel (left panel) and Western blot with pooled sera from vaccinated and control animals at T0 and T2 (right panel) with 10 µg IV proteins. MW, molecular weight markers. (B) Expression of recombinant proteins in *E. coli*. Ten µg of purified proteins were loaded per well in a 12% SDS-polyacrylamide gel. Recombinant proteins are indicated with arrows. MW, molecular weight markers. (C) Western blot analysis of recombinant mycobacterial proteins using pooled sera from IV vaccinated and control animals at T0, T1 and T2. Ten µg of purified proteins were loaded per well in a 12% SDS-polyacrylamide gel. The position of the recombinant proteins is indicated with arrows. (D) Antibody levels against mycobacterial proteins were determined by ELISA in serum samples from vaccinated and control animals and O.D._450 nm_ values (Ave+S.D.) were compared at each time point between vaccinated and control groups by Student’s t-test with unequal variance (*P≤0.05). (E, F) Correlation analysis between antibody levels against NADPAD and MPB83 mycobacterial proteins in vaccinated and control animals and (E) lesion score or (F) culture score at T2 (N = 15).

The IV was administered to boar orally in the absence of adjuvant. However, our first question focused on whether it induced an antibody response associated with protection. Western blot analysis of the IV proteins using sera from vaccinated and control animals identified proteins recognized by vaccinated animals only at T2 (bands 1–3; Fig. 2A), while other bands were recognized by vaccinated animals at T0 and T2 (band 4; Fig. 2A) or by both vaccinated and control animals (group of bands in G; Fig. 2A). Bands 1–4 were extracted from the gel and proteins identified by mass spectrometry. The results showed that band 1 corresponded to glyceraldehyde-3-phosphate dehydrogenase (GAPDH) (Genbank accession number P64179), a cell surface ligand that may be involved in mycobacterial adherence to DCs and macrophages [29]. Band 2 was identified as NADP-dependent alcohol dehydrogenase C (NADPAD) (P0A4X1) [30]. Band 3 contained two proteins, MPB83 (ACD61707) and the universal stress protein Rv2623/MT2698 (NP_337200). MPB83 is a secreted glycoprotein with homology to MPB70 [31], [32] that has been used for immunodiagnosis of *M. bovis*-infected cattle [33] and as a candidate vaccine antigen against TB [34], [35]. The universal stress protein Rv2623/MT2698 is an ATP-binding protein involved in a pathway that promotes *M. tuberculosis* persistent infection [36] and its protein sequence is identical to the *M. bovis* hypothetical protein Mb2656 (NP_856302). Band 4 was identified as MPB70 (ACD61706), a secreted protein used in ELISA for the diagnosis of cattle infected with *M. bovis* [33], [37].

The genes encoding for proteins in bands 1–3 that were identified in vaccinated animals at T2 only were cloned from the IV isolate Neiker 1403, recombinant proteins produced in *E. coli* (Fig. 2B) and used in ELISA and Western blot analysis of sera from vaccinated and control animals (Figs. 2C and 2D). The results showed that sera from control and vaccinated animals recognized *M. bovis* recombinant proteins after infection at T2, suggesting a response to infection that for most proteins was enhanced after vaccination with the IV.

Antibody titers against PPDb, DPP and GAPDH, NADPAD, MPB83, Rv2623 recombinant proteins showed differences between vaccinated and control animals for NADPAD and MPB83 at T2 only (Fig. 2D). Interestingly, although not statistically significant (P = 0.2–0.4) both NADPAD and MPB83 antibody levels had the tendency to be higher in animals with lower lesion and culture scores (Figs. 2E and 2F). These results suggested that antibodies against these proteins might be involved in the protective response elicited by the IV.

### Oral Vaccination with the IV Activates Innate Immune Response

The next question on the possible protective mechanisms associated with the IV focused on the innate immune response. Based on our previous results of differential gene expression in wild boar in response to *M. bovis* infection and vaccination and results from other groups [7], [8], [12], [14], [15], [38], [39], we selected for mRNA analysis genes involved in innate immunity (complement component 3, *C3*; NLR family, pyrin domain containing 3, *NLRP3*; Toll-like receptor adaptor molecule 1, *TRIF*; myeloid differentiation primary-response protein 88, *MYD88*; interleukin 1-beta, *IL-1b*; interferon gamma, *IFN-γ*; and interferon beta, *IFN-b*), mucosal immunity (Perforin, *PERF* and chemokine (C-C motif) receptor 7, *CCR7*), and methylmalonyl CoA mutase, *MUT* (Table 2).

**Figure 3:**
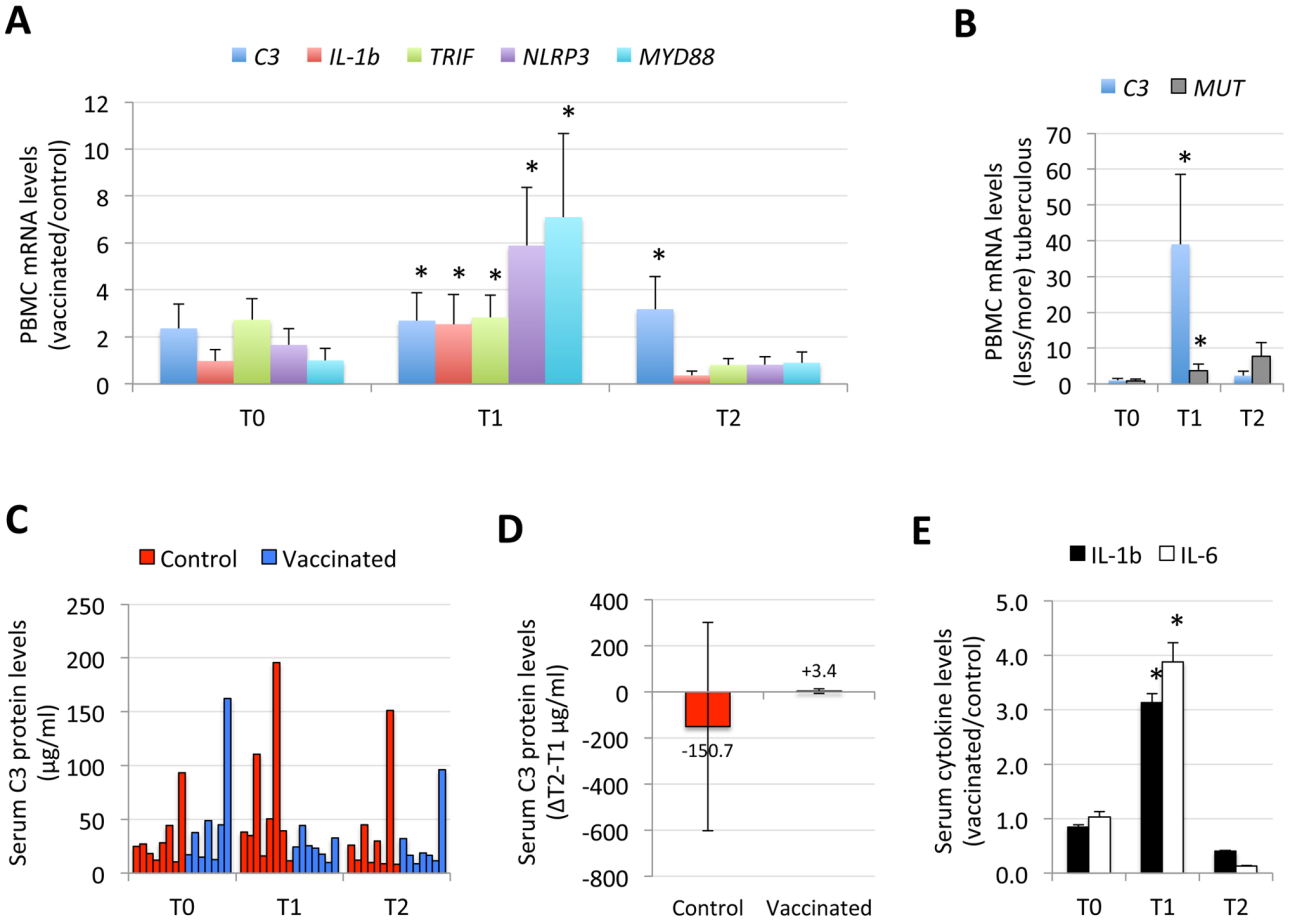
Oral vaccination with IV activates innate immune response in wild boar. (A) Comparison of PBMC mRNA levels between vaccinated (N = 7) and control (N = 8) animals. (B) Comparison of PBMC mRNA levels between control animals with lower (N = 5) and higher (N = 3) tuberculous lesion score. The mRNA levels of selected genes were analyzed by real-time RT-PCR in PBMC of vaccinated and control wild boar collected at T0 (day 1, before vaccination), T1 (day 126, before challenge) and T2 (day 255 at necropsy). The mRNA levels were normalized against *S. scrofa* cyclophilin, β-actin and GAPHD and normalized Ct values were represented as Ave+S.D. and compared between groups by Student’s t-test with unequal variance (*P≤0.05). (C) Serum C3 protein levels (µg/ml) were determined by ELISA in vaccinated and control wild boar at T0, T1 and T2 and compared between groups by Student’s t-test with unequal variance (P>0.05). (D) The difference in serum C3 protein levels (µg/ml) determined by ELISA in vaccinated and control wild boar was calculated between T2 and T1 and represented as Ave±S.D. (E) Serum cytokine protein levels were determined using the Quantibody porcine cytokine array in vaccinated (N = 7) and control (N = 8) wild boar at T0, T1 and T2, represented as Ave+S.D. and compared between groups by Student’s t-test with unequal variance (*P≤0.05).

The analysis of gene expression in wild boar PBMC showed that *C3*, *IL-1b*, *TRIF*, *NLRP3* and *MYD88* mRNA levels were higher after vaccination (T1) in vaccinated animals when compared to controls (Fig. 3A), with no effect of vaccination on *IFN-γ*, *IFN-b*, *PERF*, *CCR7* and *MUT* mRNA levels (data not shown). After infection (T2), only *C3* mRNA levels remained higher in vaccinated animals (Fig. 3A). Comparison of control animals with high (40–53; N = 3) and low (15–23; N = 5) tuberculous lesion score (Fig. 1A), showed that as in previous experiments with natural wild boar populations [13]–[15], [40], *C3* and *MUT* mRNA levels were higher in less tuberculous animals at T1 (Fig. 3B). Serum C3 protein levels did not show significant differences between vaccinated and control animals due to animal-to-animal variations (Fig. 3C), but a tendency towards increasing C3 levels after infection was observed in vaccinated but not in control animals (Fig. 3D).

Serum protein levels for pro-inflammatory cytokines IL-1b (833±44 vs. 266±11 pg/ml in vaccinated and control animals, respectively) and IL-6 (31±5 vs. 8±0 pg/ml in vaccinated and control animals, respectively) were higher in vaccinated animals at T1 (Fig. 3E), with no significant differences in the levels of IL-4, IL-8, IL-10, IL-12, GM-CSF, TGF-b1 and TNF-a (data not shown). These results together with the induction of *TRIF*, *NLRP3* mRNA levels in vaccinated animals suggested the activation of the innate immune response including inflammasome in response to the IV [39].

### The IV contains Mycobacterial DNA and RNA that Increase *C3* Expression Levels in Vaccinated Animals

The results presented here suggested inflammasome activation in response to the IV. However, this protective mechanism has been mostly associated with live and not killed vaccines [39]. Recently, prokaryotic messenger RNA was identified as viability-associated pathogen-associated molecular patterns (vita-PAMPs) involved in inflammasome activation and protection against live bacteria [39]. Although wild boar were vaccinated orally with inactivated mycobacteria, we characterized the presence and possible role of mycobacterial DNA and RNA in the IV.

**Figure 4:**
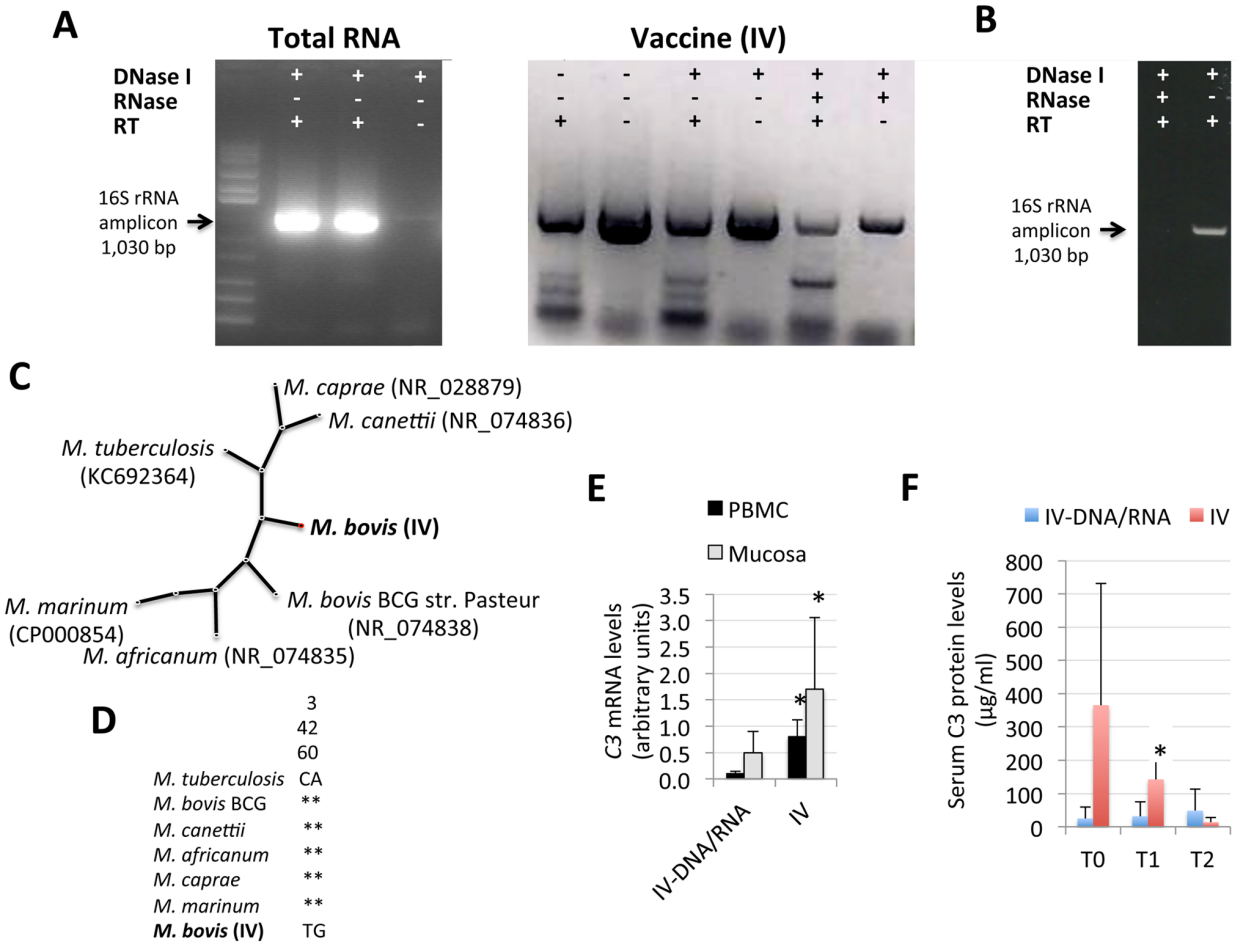
Mycobacterial DNA and RNA in the IV increase C3 expression levels. (A) Total RNA (4 µg) extracted from the IV or IV (2 µl) were subjected to different treatments and analyzed by agarose gel electrophoresis after RT-PCR for the amplification of *Mycobacterium* spp. 16S rRNA. (B) Total RNA extracted from the IV was treated with DNase I and/or RNase and used for cDNA synthesis using random primers 16S rRNA PCR. (C) Radial un-rooted tree of the 16S rRNA phylogenetic analysis using Neighbor Joining. *Mycobacterium* species and sequence Genbank accession numbers are shown. (D) Alignment of 16S rRNA sequences from the same mycobacteria used in the phylogenetic analysis to show characteristic single nucleotide polymorphisms in the vaccination isolate (*M. bovis* (IV)). (E) C3 mRNA levels in the oral mucosa and PBMC at T2. (F) Serum C3 protein levels in pigs vaccinated with the IV and IV-DNA/RNA determined by ELISA at T0, T1 and T2. The mRNA levels were normalized against *S. scrofa* cyclophilin, β-actin and GAPHD. Normalized Ct values and protein levels (µg/ml) were represented as Ave+S.D. and compared between groups by Student’s t-test with unequal variance (*P≤0.05).

The results showed that the IV contains mycobacterial DNA and RNA (Figs. 4A and 4B). Total RNA extracted from the IV was equivalent to 0.45 µg/ml (260 nm/280 nm ratio = 1.85). The sequence of the *16S rRNA* amplicon had >99% identity (E = 0.0) to other *Mycobacterium* spp. (Fig. 4C), but showed some distinctive single nucleotide polymorphisms (Fig. 4D). In a preliminary experiment, pigs were vaccinated with the IV and the IV depleted of DNA and RNA (IV-DNA/RNA). The presence of only two animals per group precluded from comparing lesion and culture scores between animals vaccinated with the IV and the IV-DNA/RNA. The analysis of *TRIF* and *NLRP3* mRNA levels did not show any difference between animals vaccinated with the IV and the IV-DNA/RNA (data not shown), suggesting that bacterial DNA/RNA were not involved in inflammasome activation in response to the IV. However, the finding of higher *C3* mRNA and serum protein levels in pigs vaccinated with the IV when compared to animals vaccinated with the IV-DNA/RNA (Figs. 4E and 4F) suggested a role for mycobacterial DNA/RNA in C3 production by a still unknown mechanism.

### IFN-γ Protein Levels Increased in Response to Oral Vaccination with the IV

Vaccination with BCG has been shown to induce protection against mycobacteria in humans, possums and mice [41]–[43] and the IFN-γ production by CD4+ T cells has been shown to play a role in protection against TB after vaccination with BCG in humans and mice [41], [42]. IFN-γ has been also shown to be involved in protection against TB in infected individuals [44]. In wild boar, IFN-γ levels increase after parenteral BCG vaccination but no correlation with protection was observed [8]. Although *IFN-γ* mRNA levels did not change in response to the IV, we next focused on this mechanism due to its possible role in protection against TB.

**Figure 5:**
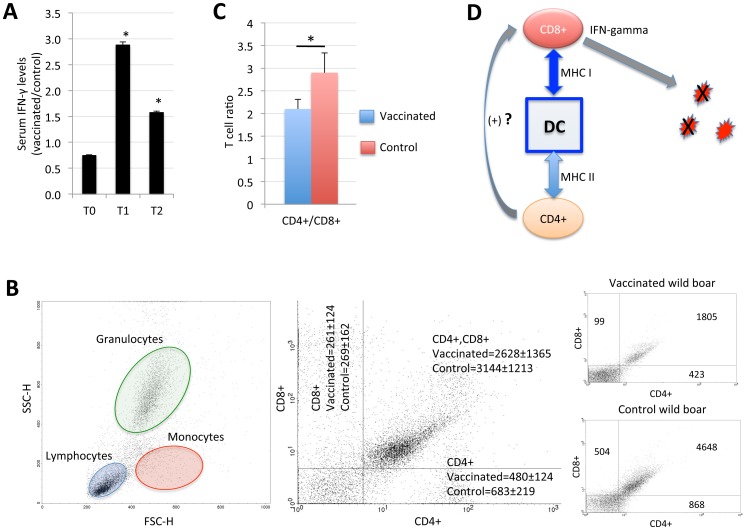
Oral vaccination with IV increases serum IFN-γ levels and CD8+/CD4+ T cell ratio. (A) Serum IFN-γ protein levels were determined using the Quantibody porcine cytokine array in vaccinated (N = 7) and control (N = 8) wild boar at T0, T1 and T2, represented as Ave+S.D. and compared between groups by Student’s t-test with unequal variance (*P≤0.05). (B) Flow citometry analysis of wild boar blood samples. Representative flow citometry profiles of blood cell populations and CD4+/CD8+ T cell counts in vaccinated and control wild boar. (C) The CD4+/CD8+ T cell ratio was determined in vaccinated (N = 7) and control (N = 8) wild boar at T1, represented as Ave+S.D. and compared between groups by Student’s t-test with unequal variance (*P≤0.05). (D) Simplified model for the generation of T cell effector responses in wild boar vaccinated with the IV. Vaccine antigens are processed and presented by DCs associated to MHC class I and/or class II molecules. CD8+ T cells recognize MHC I-peptide complexes and differentiate into IFN-γ secreting cells capable of killing infected cells or pathogens. The mechanism of CD8+ T cell differentiation mediated by CD4+ T cells may not be activated in wild boar vaccinated with the IV.

In our experiments, serum IFN-γ levels were higher in vaccinated animals at T1 and T2 when compared to controls (Fig. 5A). This result suggested that, although differences were not detected at the mRNA level, IFN-γ protein levels increased in vaccinated animals with the possible involvement of CD4+/CD8+ T cells [42], [44]. The characterization of CD4+/CD8+ T cell populations in vaccinated and control wild boar at T1 showed that the majority of the cells were CD4+/CD8+ in both vaccinated and control animals (Fig. 5B). However, the CD4+/CD8+ ratio was calculated considering CD8+/CD4− and CD8−/CD4+ cells only and showed a higher CD4+/CD8+ ratio in control animals (Fig. 5C). This result suggested a protective mechanism mediated by IFN-γ producing CD8+ T cells in response to vaccination with the IV, without a clear role for Th1 CD4+ T cells (Fig. 5D). This mechanism has been shown before in mice infected with *M. tuberculosis* [44] and recent results questioned the role previously attributed to IFN-γ production by CD4+ T cells as a protective correlate for BCG [4]. As shown before, inflammasome activation in response to the IV led to secretion of IL-1b that results in an acute inflammatory response inducing the production of downstream inflammatory cytokines such as IFN-γ [45], suggesting an additional mechanism explaining the higher IFN-γ levels in wild boar vaccinated with the IV when compared to controls.

### C3 Plays a Role in Protection Elicited by Oral Vaccination with the IV

**Figure 6:**
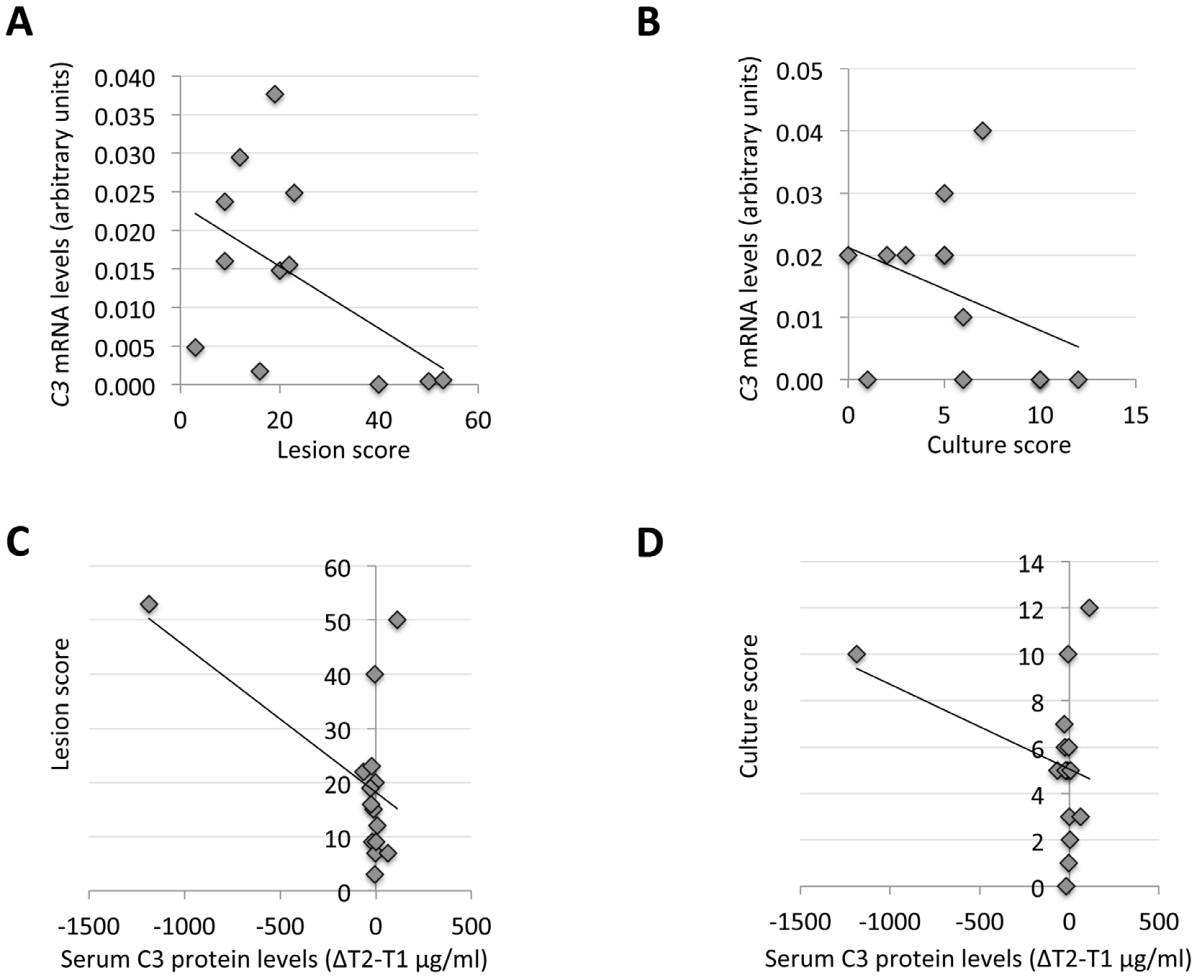
C3 mRNA and protein levels correlate with protection elicited by vaccination with the IV. (A) Correlation analysis between C3 mRNA levels in PBMC at T1 and lesion score (N = 15). (B) Correlation analysis between C3 mRNA levels in PBMC at T1 and culture score (N = 15). (C) Correlation analysis between the difference in serum C3 protein levels between T1 and T2 and lesion score (N = 15). (D) Correlation analysis between the difference in serum C3 protein levels between T1 and T2 and culture score (N = 15). The mRNA levels were normalized against *S. scrofa* cyclophilin, β-actin and GAPHD.

The results obtained here showed that C3 levels increased in response to the IV, probably regulated at the mRNA level. Correlation analysis between *C3* mRNA levels and lesion and culture scores demonstrated a tendency towards higher *C3* mRNA levels in less tuberculous animals at T1 and T2 but not at T0 (Figs. 6A and 6B and data not shown) with statistically significant correlation for lesion score (P = 0.05). The correlation between the increase in serum C3 protein levels from T1 to T2 (ΔT2−T1) and lesion and culture scores demonstrated that the increase in C3 protein levels correlated with lower lesion (P = 0.05) but not culture (P = 0.3) scores (Figs. 6C and 6D). These results together with the increase in *C3*, *IL-1b*, *TRIF* and *NLRP3* mRNA levels and IL-1b and IFN-γ protein levels in vaccinated animals, suggested that vaccination with the IV induced a protective response associated with the complement system (C3) and TRIF-mediated NLRP3 (inflammasome) activation, both involved in bacterial phagocytosis and clearance [46]–[49].

**Figure 7:**
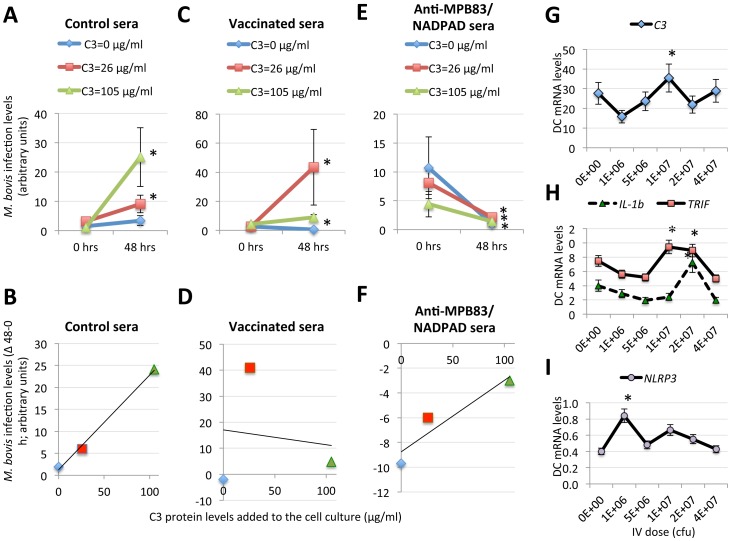
C3 plays a role in mycobacterial infection and protection elicited by DCs incubated with the IV. (A–F) Pig lung alveolar macrophages (cell line 3D4/31) were experimentally infected with *M. bovis* (SB0339 strain) at 5 mycobacteria per cell. After removing free bacteria, the cells were incubated for 48 h with 0 µg/ml, 26 µg/ml or 105 µg/ml of C3 in the presence of 10% sera from (A,B) control wild boar, (C,D) vaccinated wild boar and (E,F) rabbit IgGs raised against recombinant mycobacterial proteins MPB83 and NADPAD. DNA was used to determine mycobacterial infection levels by real-time PCR of the major immunogenic protein *MPB70*. *M. bovis* infection levels (normalized DNA Ct values) were expressed as (A,C,E) Ave±S.D. or (B,D,F) the difference between 48 and 0 h (delta 48-0 h) and compared between 0 and 48 hrs for each treatment by χ2-test (*P≤0.05; N = 2). (G–I) Pig DCs were incubated for 48 h with different amounts of the IV (0, 10^6^, 5×10^6^, 10^7^, 2×10^7^, and 4×10^7^ cfu). After incubation, cells were collected, washed with PBS and total RNA extracted for *C3*, *IL-1b*, *TRIF* and *NLRP3* gene expression analysis by real-time RT-PCR. The mRNA levels were normalized against *S. scrofa* cyclophilin, β-actin and GAPHD and normalized Ct values were represented as Ave+S.D. and compared with the group incubated without the IV by Student’s t-test with unequal variance (*P≤0.05; N = 6).

To test the role of C3 in protection elicited with the IV, an *in vitro* experiment was conducted characterizing the effect of C3 alone or in combination with anti-IV antibodies on *M. bovis* infection of cultured pig macrophages. The results showed that C3 in combination with sera from control wild boar increased infection of pig macrophages (Figs. 7A and 7B), supporting a role for the complement system in mycobacterial infection [48], [49]. However, sera from IV vaccinated wild boar inhibited infection in the absence of C3 but infection increased at higher C3 protein levels (Figs. 7C and 7D), again suggesting a role for C3 during mycobacterial infection and an inhibitory effect of anti-IV antibodies. When specific antibodies against recombinant *M. bovis* proteins recognized by IV-vaccinated wild boar were incubated in combination with C3, a reduction in mycobacterial infection was obtained (Figs. 7E and 7F). Similar results were obtained targeting by PCR the mycobacterial gene *MPB70* (Figs. 7A–7F) and of the insertion sequence IS6110 (data not shown). In summary, these results showed a role for C3 during *M. bovis* infection in the absence of antibodies against mycobacterial proteins but suggested a role for antibodies against IV proteins alone or in combination with C3 in protection against infection.

### Proposed Mechanism for the Protection Elicited by the IV against TB

After completing the experiments described before, our hypothesis was that the IV induces a protective immune response triggered by dendritic cells (DCs) mimicking phagocyte response to PAMPs [47]. To test this hypothesis, pig DCs were incubated with different IV doses to characterize the mRNA levels for genes known to be involved in cell response to PAMPs (*TRIF*, *NLRP3*, *IL-1b* and *C3*; [47], upregulated in IV-vaccinated wild boar (*TRIF*, *NLRP3*, *IL-1b* and *C3*; Fig. 4A) and which expression correlated with protective response to the IV (*C3*; Figs. 4B, 6A and 6B). The results showed that at an IV dose close to the vaccination dose (1E+07 cfu), *TRIF*, *NLRP3*, *IL-1b* and *C3* mRNA levels were higher when compared to mock treated cells (Figs. 7G–7I).

**Figure 8:**
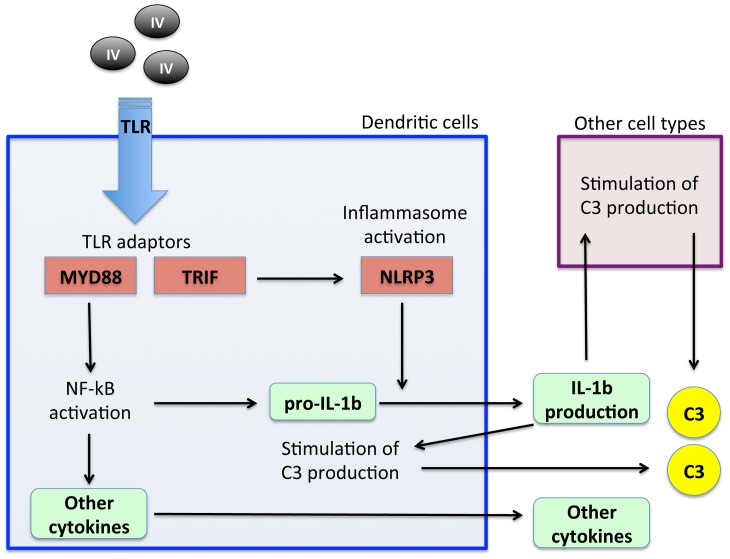
Proposed mechanism for C3 induction in response to vaccination with the IV. The IV components are sensed by dendritic cells through a surface Toll-like receptor (TLR), which triggers signaling cascades that lead to the transcription of genes encoding pro-inflammatory cytokines such as pro-IL-1β mediated by NK-kB activation through TLR adaptor, MYD88 and NLRP3 inflammasome activation promoted by TLR adaptor, TRIF. Bacterial DNA and RNA present in the IV may also induce signaling and NLRP3 inflammasome activation. NLRP3 activation converts pro-IL-1β into its bioactive mature form, which in turns stimulates the production of C3 by different cell types.

These results support a role for DCs in triggering the immune response to the IV through a mechanism similar to the phagocyte response to PAMPs (Fig. 8). After oral vaccination in the absence of adjuvants, DCs sense PAMPs present in the IV through a surface Toll-like receptor (TLR), which triggers signaling cascades that lead to the transcription of genes encoding pro-inflammatory cytokines such as pro-IL-1b and IL-6 mediated by nuclear factor kB (NF-kB) activation through TLR adaptor, MYD88 and NLRP3 inflammasome activation through TLR adaptor, TRIF by a still unknown mechanism [47]. NLRP3 activation converts pro-IL-1b into its bioactive mature form, which in turn stimulates the production of C3 by DCs and other cells types [50]–[53].

## Conclusions

In summary, C3 appears to play the central role in the protective response elicited by the oral vaccination with the IV. C3 activation is required for both classical and alternative complement activation pathways [54]. The complement system has been shown to be involved in mycobacterial pathogenesis. Mycobacteria activate complement via classical, alternative and lectin pathways [55]. *M. tuberculosis* activates the alternative complement pathway and binds C3 protein, resulting in enhanced phagocytosis by complement receptors (CR3) on human alveolar macrophages [48], [49]. This mechanism of C3 opsonophagocytosis by macrophages may result in the inhibition of bactericidal responses and pathogen survival [48], [49]. However, recently, the complement receptor CR3-mediated nonopsonic phagocytosis of mycobacteria has been proposed as the critical step for invasion and colonization of macrophages during primary infection of inhaled mycobacteria [56]–[58].

The upregulation of *C3* in lymph nodes and tonsils correlates with resistance to TB in wild boar [12], [14], [15]. As shown here, this mechanism may be enhanced by vaccination with the IV to protect animals against mycobacterial infection [7], [8]. *M. tuberculosis* acquire opsonic C3 peptides by at least two distinct mechanisms and can bind to CR3 at two distinct sites on the receptor [59]. Opsonized *M. tuberculosis* binds CR3 at its C3bi binding domain, and nonopsonized *M. tuberculosis* uses its endogenous capsular polysaccharides to interact with the β-glucan binding site near the C-terminus of CD11b [59]. Higher C3 levels may allow increased opsonophagocytosis and effective bacterial clearance, while interfering with CR3-mediated opsonic and nonopsonic phagocytosis of mycobacteria, a process that could be enhanced by specific antibodies against mycobacterial proteins and/or lipids induced by vaccination with the IV [55]. Additionally, the activation of IFN-γ producing CD8+ T cells by MHC I antigen presenting DCs may also contribute to the protection elicited by the IV in wild boar.

These results suggest that the IV acts through novel mechanisms to protect against TB in wild boar. However, additional experiments are required to fully characterize these protective mechanisms. Some of these mechanisms may be common to different bacterial strains while others could be associated with genetic signatures present in the bacterial genome of the IV strain [5]. Finally, the impact of the IV for TB control in wild boar needs to be demonstrated in field trials to consider possible applications in other reservoir hosts and humans. If proven effective for TB control, vaccination with the IV would have some advantages over BCG or other live vaccines such as higher stability under field conditions and safety.
